# Fibroblast growth factor 21 (FGF21) is a sensitive marker of osteoporosis in haemodialysis patients: a cross-sectional observational study

**DOI:** 10.1186/s12882-021-02393-z

**Published:** 2021-05-19

**Authors:** Lili Zhu, Min Li, Qianqian Zha, Min Yang, Jirong Yu, Mingming Pan, Qing Yin, Liqiong Jiang, Meixia Xia, Bi-Cheng Liu, Bin Wang

**Affiliations:** 1 Institute of Nephrology, Zhong Da Hospital, Southeast University School of Medicine, Nanjing, Jiangsu China; 2 Institute of Nephrology, the Third Affiliated Hospital of Soochow University, Changzhou, China

**Keywords:** Osteoporosis, Fibroblast growth factor 21, CT attenuation values, Haemodialysis, CKD-MBD

## Abstract

**Introduction:**

Osteoporosis is one of the important bone abnormalities in chronic kidney disease-mineral and bone disorder (CKD-MBD) and still lacks a sensitive biomarker to diagnose. Fibroblast growth factor 21 (FGF21) can stimulate bone loss in patients with diabetes and increase in CKD patients. In this study, we investigated whether FGF21 could serve as a biomarker to predict osteoporosis in a haemodialysis cohort.

**Methods:**

We recorded demographic information, biochemical data, and serum FGF21 and FGF23 levels and measured the CT attenuation values of 339 haemodialysis patients from two large medical centres. We assessed the correlation of CT attenuation values with serum FGF21 and FGF23 levels and tested whether they were independent factors for osteoporosis. ROC curves were constructed to compare the prognostic value of FGF21 and FGF23 for osteoporosis.

**Results:**

Based on the CT attenuation value, serum FGF21 levels were higher in our osteoporosis group (median 640.86 pg/ml vs. 245.46 pg/ml, *P* ˂ 0.01). Meanwhile, FGF21 (*r* = -0.136, *P* < 0.05) and FGF23 (*r* = -0.151, *P* < 0.05) were both negatively associated with osteoporosis. Moreover, FGF21 (*β* = -0.067, *P* < 0.05) was an independent factor for osteoporosis. Furthermore, FGF21 combined with age yielded a marked specificity (90.5 %) and sensitivity (61.8 %) in predicting osteoporosis of haemodialysis patients with less residual renal function.

**Conclusions:**

FGF21 has a positive relationship with the incidence of osteoporosis in patients on haemodialysis. FGF21 combined with age is a good predictive biomarker for osteoporosis in patients on haemodialysis, especially those with less residual renal function.

**Supplementary Information:**

The online version contains supplementary material available at 10.1186/s12882-021-02393-z.

## Introduction

Chronic kidney disease-mineral and bone disorder (CKD-MBD), a systemic disorder of mineral and bone metabolism [1], has a high prevalence and morbidity in patients with CKD [2], especially in patients with end-stage renal disease (ESRD) [3]. CKD-MBD is composed of three main aspects: renal bone disease, vascular or other soft-tissue calcification, and biochemical abnormalities such as calcium, phosphate, parathyroid hormone (PTH), and fibroblast growth factor 23 (FGF23) [4]. Osteoporosis characterized by low bone mineral density and reduced mechanical strength [5] is the most clinically relevant feature of renal bone disease. As reported, individuals with early stages of CKD may have a higher prevalence of osteoporosis and fracture risk compared to non-CKD and 20 % mortality from hip fractures within the first year [6–8]. Many factors, such as secondary hyperparathyroidism, vitamin D deficiency, increased FGF23, and metabolic acidosis, are involved in the pathophysiology of subsequent osteoporosis in patients with CKD [9].

The FGF19 subfamily, composed of FGF19, FGF21, and FGF23, is involved in diverse metabolic regulation of interorgan endocrine signalling axes [10]. FGF23 is a 32-kDa glycoprotein secreted by osteocytes and functions in calcium and phosphorus metabolism disorders [11], bone loss [12], vascular calcification [13] and other CKD-MBD events in patients with ESRD. In previous studies, some scholars proposed that FGF23 could predict bone loss in patients with ESRD through its strong correlation with bone mineral density (BMD) in lumbar spine sites and femoral neck sites [14, 15]. However, Wohlfahrt P and Isakova T et al. drew contradictory conclusions that FGF23 had no correlations with BMD and bone mass corrected for other factors such as height, eGFR and PTH levels [16, 17]. Therefore, the role of FGF23 in the changes in BMD in patients with CKD is still controversial. FGF21, another member of the FGF19 subfamily, is a powerful regulator of glucose and lipid metabolism and is mainly expressed in the liver [18, 19]. FGF21 is excreted primarily by the kidney, and its level increases markedly in patients with impaired renal function [20]. In animal models, genetic FGF21 overexpression decreases bone mass, and systemic FGF21 treatment leads to severe bone loss [21]. Meanwhile, FGF21 can accelerate bone loss by potentiating the effects of peroxisome proliferator-activated receptor γ [21]. Moreover, higher FGF21 levels synthesized in the liver can induce insulin-like growth factor binding protein 1 (IGFBP1) to stimulate osteoclast differentiation and bone resorption [18]. However, the association of FGF21 with bone loss in CKD has rarely been explored in previous studies.

In the present study, we evaluated the relationship between FGF21 and FGF23 and osteoporosis in a population-based retrospective HD cohort. First, we compared the serum FGF21 and FGF23 levels in patients on HD with or without osteoporosis on the basis of CT attenuation values. Second, we clarified whether FGF21 and FGF23 were independently associated with CT attenuation values. Ultimately, we explored the predictive values of serum FGF21 and FGF23 on osteoporosis in our HD cohort.

## Materials and methods

### Study design and subjects

**Fig. 1:**
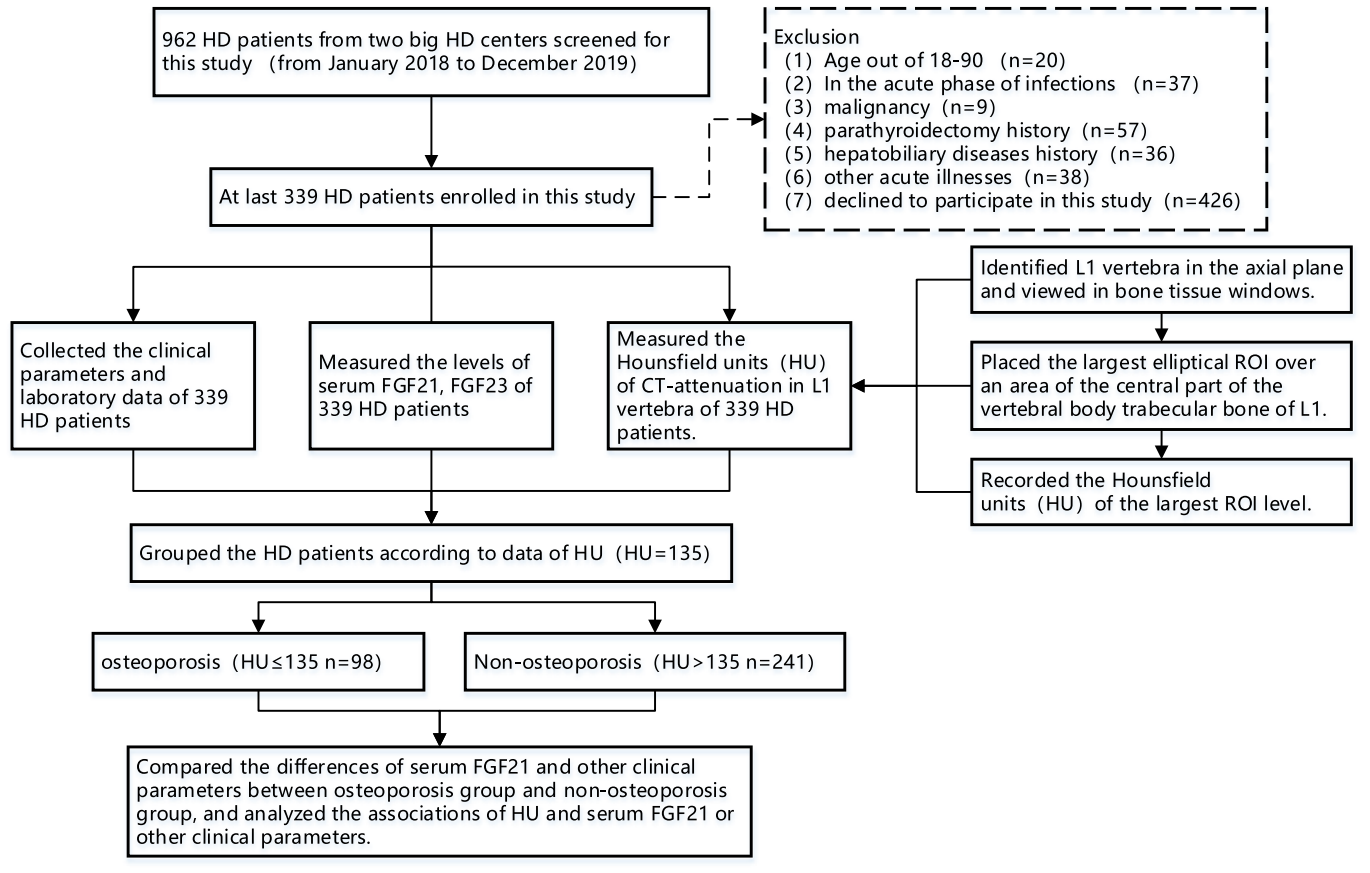
Flow chart of the study process. HD, haemodialysis; FGF21, fibroblast growth factor 21; FGF23, fibroblast growth factor 23

This was a cross-sectional observational study composed of 339 patients on HD from two large haemodialysis centres, Nanjing Zhongda Hospital and the First People’s Hospital of Changzhou, from January 2018 to December 2019 (Fig. 1). The exclusion criteria were as follows: (1) age not within 18 to 90 years old, (2) acute phase of infections, (3) malignancy, (4) parathyroidectomy history, (5) hepatobiliary disease history, (6) other acute illnesses, and (7) decline to participate in this study. All enrolled patients had received regular dialysis (3 ~ 4 times per week) for at least one month. The study protocol was approved by the Ethics Committee of Zhongda Hospital affiliated with Southeast University, and the study was conducted in accordance with the Helsinki Declaration and Chinese law. The details of the study were explained to every patient; a signed informed consent was obtained from all subjects. We confirmed that all methods were carried out in accordance with relevant guidelines and regulations.

### Clinical and biochemical data

Blood samples containing haemoglobin (Hb), serum albumin (Alb), uric acid, calcium (Ca), phosphate (Pi), total cholesterol (TC), triglyceride (TG), dicarbonate and intact parathyroid hormone (iPTH) were collected before dialysis and measured via routine laboratory methods. Serum iPTH was measured by Electrochemiluminescence Technology (Cobase411 analyser, Germany, Roche Diagnostics®). This method is an immunological test for the quantitative determination of iPTH. The interassay coefficient of variation for iPTH was lower than 4 %. We also recorded the comorbidities (hypertension, diabetes mellitus and cardiocerebrovascular disease [CVD]) and medical usages (calcium supplements, vitamin D, ACEI/ARB and cinacalcet) for every individual by checking their medical records.

### Measurement of serum FGF21 and FGF23 in patients on HD

Blood samples were collected under the fasting overnight and before dialysis. They were withdrawn in vacuum tubes and then centrifuged at 3000 rpm for 10 min. The upper serum was collected and stored at -80 °C immediately for future analyses. Serum FGF21 and FGF23 levels were assessed by enzyme-linked immunosorbent assay (ELISA) kits for human FGF21 (Neobioscience, China) and for human FGF23 (Joyee biotechnics, China), respectively. The interassay and intraassay coefficients of variation were both less than 10 % for FGF21 and FGF23.

### Attenuation assessment on CT scans

**Fig. 2:**
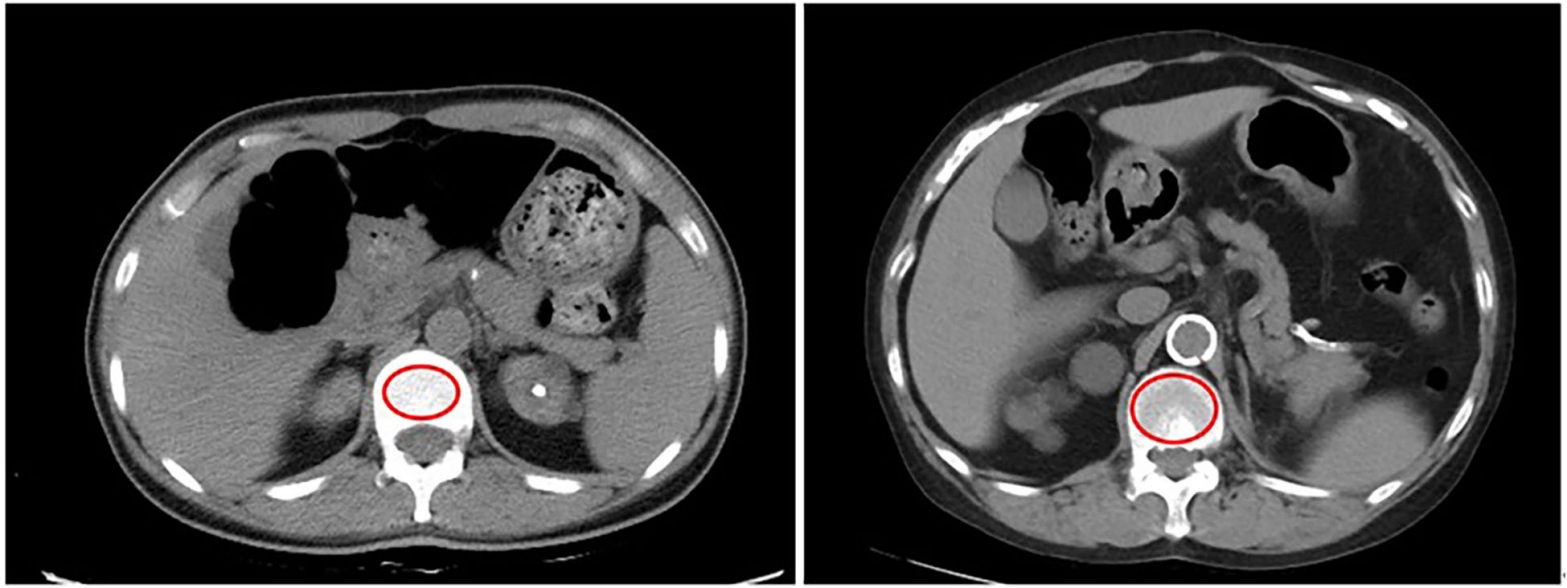
Axial image through the vertebral body of L1 on a thoracoabdominal-pelvic CT scan. Placement of the region of interest within the trabecular bone and assessment of the CT attenuation value in Hounsfield units (182.3 HU left and 96.5 HU right in this example)

Discovery HD 750 64-slice CT (GE Health care, USA) was used to measure osteoporosis. We identified the L1 vertebra in the axial plane and viewed it in bone tissue windows. Then, we placed the largest elliptical ROI on an area of the central part of the vertebral body trabecular bone of L1 to measure the vertebral BMD. In this process, the cortical margins should be excluded to prevent volume averaging, as shown in Fig. 2. All CT scans were performed by a single observer and the same CT machine to ensure consistency of regions. The mean CT attenuation values for each patient were measured in HU. We defined CT attenuation values less than 135 HU as osteoporosis [22].

### Statistical analyses

Statistical analyses were performed using SPSS 18.0 software. The data are given as the mean ± SD for normally distributed variables and as the median and interquartile range for non-normally distributed variables. Univariate analyses were performed to compare the differences between two groups. Meanwhile, we used bivariate correlation analyses to assess the correlation of CT attenuation with serum FGF21 and other clinical parameters. We employed stepwise multivariate linear regression analyses to evaluate the independent association of variables with L1 attenuation. ROC curves were constructed to calculate the AUC and compared the prognostic value of every independently associated factor or group of factors to osteoporosis. All analyses were two-tailed, and a *P* value < 0.05 was considered to be statistically significant.

## Results

### General characteristics of subjects

In this cross-sectional study, of the 962 patients on HD screened, a total of 339 patients were eligible (193 men, 56.5 %) with a mean (SD) age of 56.79 (15.60) years (Table 1). In the process of data collection, 89 patients lack blood data and 124 patients lack CT scans, we excluded 213 patients for the accuracy of our study. The dialysis duration was 4.33 ± 4.91 years, and the body mass index was 22.99 ± 4.01 kg/m^2^. According to the threshold of osteoporosis (CT attenuation values ≤ 135 HU) proposed by Pickhardt et al. [22], 98 (28.9 %) patients were enrolled in the osteoporosis group, and 241 (71.1 %) were enrolled in the non-osteoporosis group. The basic characteristics of all patients divided into these two groups are presented in Table 1. In the osteoporosis group, patients on HD had a mean age of 66.45 ± 13.28 years and a median serum FGF21 level of 640.86 pg/ml (interquartile range 1.72, 3176.14). In the non-osteoporosis group, patients had a mean age of 52.86 ± 14.77 years and a median serum FGF21 level of 245.46 pg/ml (interquartile range 0.95, 1764.29). As shown in Table 1, there were significant differences in age, diastolic blood pressure (DBP), and vitamin D usage rate between these two groups (all *P* < 0.05). Serum FGF21 levels were significantly higher in the osteoporosis group (*P* < 0.001), while serum FGF23 levels were not significantly different between these two groups. Additionally, the percentage of patients who suffered diabetes and cardiocerebrovascular disease (CVD) in the osteoporosis group was higher than that in the non-osteoporosis group (*P* < 0.001). The baseline characteristics based on the FGF21 median were shown in Table 2. Compared to low serum FGF21group, hemoglobin, albumin and dicarbonate levels were all significantly lower in high serum FGF21 level group (*P* < 0.05). Meanwhile, the proportion of HD patients with hypertension in high serum FGF21 level group is less than that in low serum FGF21 group (*P* < 0.05). However, the CT attenuation had no statistical difference between the two groups.

**Table 1:** Comparison of clinical characteristics and laboratory data between the osteoporosis and non-osteoporosis groups in patients on HD

	All HD patients	osteoporosis	non-osteoporosis	*P* value
**Number**	339	98	241	
***General data***				
Age(years)	56.79 ± 15.60	66.45 ± 13.28	52.86 ± 14.77	< 0.001**
Sex(male %)	193(56.93 %)	60(61.22 %)	133(55.19 %)	0.309
Dialysis vintage(years)	4.33 ± 4.91	4.63 ± 4.87	4.21 ± 4.94	0.469
Body mass index(BMI)	22.99 ± 4.01	22.93 ± 4.03	23.01 ± 4.01	0.868
Systolic BP(mmHg)	145.78 ± 24.40	145.94 ± 24.37	145.72 ± 24.46	0.940
Diastolic BP(mmHg)	82.32 ± 14.60	78.51 ± 14.55	83.87 ± 14.36	0.002**
***Blood data***				
Hemoglobin(g/L)	97.57 ± 20.14	98.47 ± 20.94	97.20 ± 19.84	0.601
Albumin(g/L)	35.89 ± 6.13	35.36 ± 6.41	36.11 ± 6.02	0.309
Uric acid(mmol/L)	378.64 ± 125.83	368.83 ± 120.70	382.63 ± 127.89	0.361
Triglycerides(mmol/L)	1.81 ± 1.37	1.75 ± 1.45	1.84 ± 1.33	0.438
Total cholesterol(mmol/L)	4.13 ± 1.12	4.06 ± 1.00	4.16 ± 1.17	0.605
Dicarbonate(mmol/L)	22.65 ± 3.76	22.63 ± 3.87	22.66 ± 3.72	0.943
Calcium(mmol/L)	2.26 ± 0.24	2.28 ± 0.25	2.26 ± 0.24	0.548
Phosphate(mmol/L)	1.70(0.19,4.16)	1.69(0.28,4.16)	1.76(0.19,3.35)	0.305
Parathormon(pg/ml)	374.07 ± 418.87	347.02 ± 385.80	385.07 ± 431.87	0.449
FGF21 quartiles (pg/ml)	386.67	930.23	288.80	< 0.001**
FGF23 quartiles (pg/ml)9309.83 ± 10410.899529.83 ± 9485.399220.37 ± 10782.060.804	13116.71	16210.65	13189.01	0.804
***Comorbidity***				
Diabetes(%)	112(33.04 %)	45(45.92 %)	67(27.80 %)	0.001**
Hypertension(%)	298(87.91 %)	87(88.78 %)	211(87.55 %)	0.754
CVD(%)	94(27.73 %)	48(48.98 %)	46(19.09 %)	< 0.001**
***Medicine usage***				
Vitamin D(%)	151(44.54 %)	31(31.63 %)	120(49.79 %)	0.002**
Calciu supplements(%)	86(25.37 %)	22(22.45 %)	64(26.56 %)	0.431
Cinacalcet(%)	52(15.34 %)	11(11.22 %)	41(17.01 %)	0.180
ACEI/ARB(%)	94(27.73 %)	28(28.57 %)	66(27.39 %)	0.825
***FRAX(306)***^***a***^				
Major Osteoporosis	3(0.9,14)	4.0(1.0,13.0)	2.55(0.9,14)	< 0.001**
Hip fracture	0.8(0.0,10.0)	1.95(0,10)	0.5(0,10)	< 0.001**

The values are shown as the mean ± SD, median (interquartile range) or numbers (%), *P<0.05, **P<0.01.

^a^306 of 339 patients agreed to complete the FRAX questionnaire.

*HD* haemodialysis; *BP* blood pressure; *FGF21* fibroblast growth factor 21; *FGF23* fibroblast growth factor 23; *CVD* cardiocerebrovascular disease; *ACEI* angiotensin-converting enzyme; *ARB* angiotensin receptor II antagonist; *FRAX* fracture risk assessment tool

**Table 2:** Comparison of clinical characteristics and laboratory data between the low serum FGF21 level group and high serum FGF21 level group in patients on HD

	Low FGF21 group ( FGF21 ≤ 184.50pg/ml )	High FGF21 group ( FGF21>184.50pg/ml )	*P* value
**Number**	170	169	
***General data***			
Age(years)	55.82 ± 15.60	57.76 ± 15.59	0.253
Sex(male %)	100(58.82 %)	93(55.03 %)	0.481
Dialysis vintage(years)	4.17 ± 4.45	4.49 ± 5.35	0.540
Body mass index(BMI)	22.93 ± 3.93	23.05 ± 4.09	0.794
Systolic BP(mmHg)	145.75 ± 24.91	145.82 ± 23.95	0.979
Diastolic BP(mmHg)	82.39 ± 13.95	82.25 ± 15.27	0.933
***Blood data***			
Hemoglobin(g/L)	99.92 ± 19.80	95.21 ± 20.07	0.031*
Albumin(g/L)	36.80 ± 6.39	34.98 ± 5.74	0.006**
Uric acid(mmol/L)	379.78 ± 134.65	377.50 ± 116.68	0.868
Triglycerides(mmol/L)	1.75 ± 1.39	1.88 ± 1.35	0.412
Total cholesterol(mmol/L)	4.19 ± 1.11	4.08 ± 1.13	0.346
Dicarbonate(mmol/L)	23.06 ± 3.74	22.24 ± 3.74	0.044*
Calcium(mmol/L)	2.27 ± 0.24	2.26 ± 0.24	0.639
Phosphate(mmol/L)	1.73 ± 0.56	1.75 ± 0.61	0.744
Parathormon(pg/ml)	369.08 ± 452.80	379.08 ± 383.00	0.826
***CT***			
L1 attenuation (HU)	168.90 (137.75, 197.00)	159.10 (118.15, 212.00)	0.363
***Comorbidity***			
Diabetes(%)	60(35.29 %)	50(29.59 %)	0.262
Hypertension(%)	155(91.18 %)	141(83.43 %)	0.032*
CVD(%)	43(25.29 %)	34(20.12 %)	0.255
***Medicine usage***			
Vitamin D(%)	76(44.71 %)	75(44.38 %)	0.952
Calciu supplements(%)	41(24.12 %)	45(26.63 %)	0.595
Cinacalcet(%)	31(18.24 %)	21(12.43 %)	0.138
ACEI/ARB(%)	52(30.59 %)	42(24.85 %)	0.238
***FRAX(306)***^***a***^			
Major Osteoporosis	3.78 ± 2.56	3.64 ± 2.70	0.637
Hip fracture	1.61 ± 1.85	1.57 ± 2.04	0.881

The values are shown as the mean ± SD, median (interquartile range) or numbers (%), *P<0.05, **P<0.01.

^a^306 of 339 patients agreed to complete the FRAX questionnaire.

*HD* haemodialysis; *BP* blood pressure; *FGF21* fibroblast growth factor 21; *HU* hounsfield unit; *CVD* cardiocerebrovascular disease; *ACEI* angiotensin-converting enzyme; *ARB* angiotensin receptor II antagonist; *FRAX* fracture risk assessment tool

### Correlations of CT attenuation values with FGF21 and FGF23 in HD patients

**Fig. 3:**
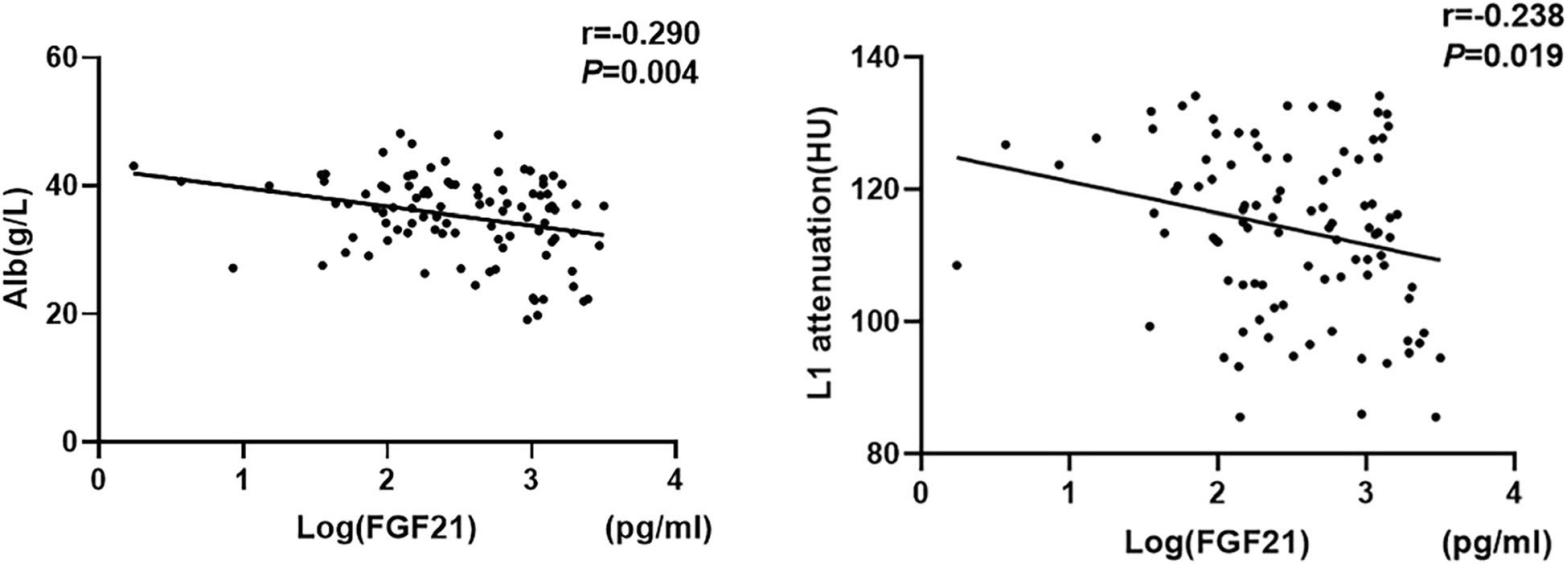
Correlation of serum FGF21 and albumin and L1 attenuation in osteoporosis group of HD patients. Log (FGF21) was significantly correlated with (a) albumin (*r* = -0.290, *P* < 0.05); (b) L1 attenuation (*r* = -0.238, *P* < 0.05). FGF21, fibroblast growth factor 21; Alb, albumin

In all patients on HD, serum FGF21 and FGF23 levels were both negatively related to CT attenuation values (FGF21: *r*_*1*_=-0.136, *P*_*1*_ = 0.012; FGF23: *r*_*2*_=-0.151, *P*_*2*_ = 0.005). In our osteoporosis group, CT attenuation and Log (FGF21) were normally distributed data and CT attenuation values were also negatively associated with Log (FGF21) (*r* = -0.238, *P* = 0.019) in pearson correlation analysis (Fig. 3). Univariate logistic regression analysis also showed that CT attenuation is negatively associated with above-tertile serum FGF21 levels (odds ration [OR] = 0.420, *P* < 0.001) (Supplemental Table 1). In addition, CT attenuation values were negatively correlated with age (*r* = -0.440, *P* < 0.001) and dialysis duration (*r* = -0.120, *P* = 0.028), while the CT attenuation values were positively correlated with DBP (*r* = 0.274, *P* < 0.001), as shown in Table 3.

**Table 3:** Bivariate correlation analyses for the correlations of L1 CT attenuation with serum FGF21 levels and other variables in patients on HD

Variables																
	Age		Dialysis	SBP	DBP	Hb	Alb	TG	Ca	Pi	iPTH	FGF21	FGF23		MO	HF
**r**	-0.440		-0.120	0.030	0.274	-0.019	-0.027	0.017	-0.022	0.089	0.105	-0.136	-0.151		-0.383	-0.411
***P* value**	< 0.001**		0.028*	0.580	< 0.001**	0.721	0.623	0.761	0.692	0.104	0.053	0.012*	0.005**		< 0.001**	< 0.001**

*P<0.05, **P<0.01

*FGF21* fibroblast growth factor 21; *FGF23* fibroblast growth factor 23; *SBP* systolic blood pressure; *DBP* diastolic blood pressure; *Hb* haemoglobin; *Alb* serum albumin; *Ca* calcium; *Pi* phosphate; *TG* triglyceride; *iPTH* intact parathyroid hormone; *MO* major osteoporosis; *HF* hip fracture

### Multivariate linear regression analyses for independently associated factors of CT attenuation values and serum FGF21 levels

Those parameters that were different between the osteoporosis and non-osteoporosis groups and well-known risk factors, such as dialysis duration, SBP, Hb, Alb, TG, Ca, Pi, iPTH and FGF23, were all included in multivariate linear regression analyses. As shown in Table 4, only FGF21 (*β* = -0.067, *P* < 0.05), age (*β* = -1.362, *P* < 0.05) and DM (*β* = -27.013, *P* < 0.05) were independently and negatively associated with CT attenuation in patients on HD. Meanwhile, albumin (*β* = -0.062, *P* < 0.001), dicarbonate (*β* = -0.079, *P* < 0.001), calcium (*β* = 0.796, *P* < 0.05), CT attenuation (*β* = -0.519, *P* < 0.05) and comorbidity of hypertension (*β* = -0.575, *P* < 0.05) were independently associated with serum FGF21 levels in patients on HD, as shown in Table 5. In multivariate logistic regression analysis, CT attenuation is positively associated with age (OR = 1.065, *P* < 0.001) and FGF21 levels (OR = 1.002, *P* < 0.001) (Supplemental Table 2).

**Table 4:** Multivariate linear regression analyses for the establishment of factors independently associated with CT attenuation

Variables	β(95 % CI)	*P* value
Age	-1.021(-2.212,-0.512)	<0.001**
Dialysis duration	-0.040	0.467
BMI	-0.064	0.192
SBP	-0.042	0.547
DBP	0.692(0.256, 1.127)	0.002**
TG	-0.098	0.079
TC	-0.093	0.094
Ca	0.005	0.923
Pi	-0.085	0.148
iPTH	0.044	0.429
FGF21	-0.067(-0.104,-0.030)	0.019*
FGF23	-0.012	0.832
CVD	-0.084	0.147
DM	-18.029(-31.217,-4.841)	0.008*
Ca usage	-0.041	0.480
Vitamin D usage 0.044		0.451

*P<0.05, **P<0.01

*FGF21* fibroblast growth factor 21; *FGF23* fibroblast growth factor 23; *TC* total cholesterol; *TG* triglyceride; *Ca* calcium; *Pi* phosphate; *iPTH* intact parathyroid hormone; *CVD* cardiocerebrovascular disease; *DM* diabetes mellitus

**Table 5:** Multivariate linear regression analyses for the establishment of factors independently associated with FGF21

Variables	β(95 % CI)	*P* value
Age	0.092	0.089
Dialysis duration	0.065	0.240
BMI	0.013	0.799
SBP	0.077	0.138
DBP	0.093	0.082
TG	-0.053	0.309
TC	0.055	0.296
Ca	0.796(0.111,1.482)	0.023*
Pi	0.005	0.924
iPTH	0.000	0.994
Alb	-0.062(-0.088,-0.035)	< 0.001**
Dicarbonate	-0.079(-0.121,-0.038)	< 0.001**
CVD	-0.048	0.362
DM	-0.019	0.726
HBP	-0.575(-1.020,-0.130)	0.011*
Ca usage	0.039	0.460
Vitamin D usage -0.005		0.922

*P<0.05, **P<0.01

*FGF21* fibroblast growth factor 21; *BMI* body mass index; *TC* total cholesterol; *TG* triglyceride; *Ca* calcium; *Pi* phosphate; *iPTH* intact parathyroid hormone; *Alb* albumin; *CVD* cardiocerebrovascular disease; *DM* diabetes mellitus; *HBP* high blood pressure

### Prediction by FGF21 and FGF23 of osteoporosis in patients on HD

In this study, receiver operating characteristic (ROC) curves were constructed to assess distinguished values of independently associated factors in predicting osteoporosis (Table 6). The area under the curve (AUC) of FGF21 in predicting osteoporosis was 0.71 (95 % *CI*, 0.64 to 0.78, *P* < 0.001), with good sensitivity (80.2 %) but low specificity (41.1 %). Notably, the AUC of FGF21 combined with age in predicting osteoporosis increased significantly to 0.829 (95 % *CI*, 0.78 to 0.88, *P* < 0.001) with optimal sensitivity (91.4 %) and higher specificity (47.9 %). In the two subgroups based on 24-hour urine volume in HD patients, the area under the curve (AUC) of FGF21 in predicting osteoporosis were 0.657 (95 % *CI*, 0.558 to 0.755, *P* = 0.001) and 0.691 (95 % *CI*, 0.606 to 0.775, *P* = 0.001), respectively (Table 6). FGF21 has a good specificity (86.7 % vs. 92.1 %) but low sensitivity (44.2 % vs. 41.8 %) in two subgroups. In HD patients with 24-hour urine volume **>** 100ml, the AUC of FGF21 combined with age in predicting osteoporosis was 0.837 (95 % *CI*, 0.778 to 0.896, *P* < 0.001) with better sensitivity (70.1 %) and specificity (81.4 %). In HD patients with 24-hour urine volume **≤** 100ml, the AUC of FGF21 combined with age in predicting osteoporosis was 0.833 (95 % *CI*, 0.772 to 0.894, *P* < 0.001) with good sensitivity (61.8 %) and specificity (90.5 %).

**Table 6:** The area under the curve (AUC) of separated and grouped independently associated factors of L1 CT attenuation in ROC curve analyses in HD patients

Variables	All HD patients		HD patients with anuria		HD patients without anuria	
	**AUC(95 %*CI*)**	***P*value**	**AUC(95 %*CI*)**	***P*value**	**AUC(95 %*CI*)**	***P*value**
Age	0.712 (0.644,0.779)	< 0.001**	0.756 (0.681,0.832)	< 0.001**	0.754 (0.685,0.822)	< 0.001**
FGF21	0.710 (0.638,0.781)	< 0.001**	0.657 (0.558,0.755)	0.001**	0.691 (0.606,0.775)	< 0.001**
Age + FGF21	0.829 (0.776,0.882)	< 0.001**	0.837 (0.778,0.896)	< 0.001**	0.833 (0.772,0.894)	< 0.001**

*P<0.05, **P<0.01

*FGF21* fibroblast growth factor 21; *HD* haemodialysis

## Discussion

In the present study, we showed that serum FGF21 levels were significantly higher in the osteoporosis group of patients on HD. FGF21 rather than FGF23 is independently and negatively associated with CT attenuation values in patients on HD. Furthermore, FGF21 combined with age showed good specificity (90.5 %) and sensitivity (61.8 %) for the prediction of osteoporosis in patients on HD with less residual renal function.

Many previous researches have showed the values of L1 trabecular attenuation at routine CT can identify the risk of osteoporosis in general population and the most optimized threshold was 135 HU [22, 23]. In our study, we found that the mean age of the osteoporosis group was higher and that CT attenuation values declined with ageing in bivariate correlation analyses, which is in line with the characteristic of osteoporosis that the bone mass reaches its highest level in adolescence and then is subsequently lost with ageing [24]. In addition, we found that the percentage of vitamin D supplements was higher in the non-osteoporosis group, which may be due to the protective function of vitamin D on improving osteoporosis by affecting the number of osteoblasts, osteoclasts and osteocytes in bone [25].

FGF23 is a recently identified hormone that is produced in bone by osteocytes and osteoblasts [26]. In previous studies, FGF23 suppressed bone mineralization by inhibiting cell differentiation, number, activity and bone turnover [27–29]. In animal models, the role of FGF23 in regulating phosphate metabolism can be one causative factor of abnormal bone mineralization [30, 31]. Our result that the FGF23 level was negatively associated with CT attenuation values was potent evidence for its role in bone loss as well. However, our results indicated that FGF23 was not an independent risk factor for osteoporosis (Table 3), and it had no value in predicting osteoporosis in this study (data not shown), even though Mirza Ma and Lane Ne et al. demonstrated that serum FGF23 levels were related independently to fracture risk in elderly men with decreased estimated glomerular filtration (eGFR) [32, 33]. These discrepancies may be due to the different methods used to measure bone mineral density, as dual-energy X-ray absorptiometry (DXA) was mostly used in previous studies.

FGF21 is another member of the FGF19 subfamily that is primarily secreted from the liver under physiological conditions and other sites, including adipose tissue, skeletal muscle, heart and kidney [34]. Multiple studies have shown that high-levels of serum FGF21 have association with the loss of bone mineral density in adults and postmenopausal women with normal renal function [35, 36]. Moreover, its level increased progressively with a decline in renal function, as renal clearance is considered to be the main route of FGF21 excretion [20]. Interestingly, we found that serum FGF21 levels were higher in our osteoporosis group than in the non-osteoporosis group in patients on HD (Table 1). Of note, our results also indicated that FGF21 was an independent risk factor for osteoporosis in multivariate linear regression analyses. Consistent with our results, a recent study conducted in patients undergoing haemodialysis also showed that serum FGF21 level was significantly negatively associated with BMD despite evaluated by DXA [37]. In addition to HD cohort, the relationship between FGF21 and bone mineral density was observed in normal kidney function as well [38]. The relevant mechanism could be explained by the following facts: (1) physiological or pharmacological elevation of FGF21 induced IGFBP1 expression, which could be secreted into circulation and stimulate osteoclast differentiation, bone resorption and reduce bone mass [18]; (2) a previous study showed that IGFBP1 was significantly increased in patients on HD compared to healthy controls [35], which is consistent with our result that IGFBP1 levels are nearly eight times higher in the osteoporosis group than in the non-osteoporosis group (data not shown); and (3) FGF21 functions to trigger intracellular calcium release [39] and increase phosphate intake [40], which is in line with our result that FGF21 had a positive relationship with serum calcium levels in this study (Table 5). Different from the role of FGF23 in regulating calcium and phosphorus levels, FGF21 is mainly involved in bone metabolism by influencing bone cells formation. Of course, the causative effect of FGF21 on osteoporosis in patients on HD and the exact mechanism of the involvement of FGF21 in the regulation of osteoporosis need to be further confirmed by large-sample RCT studies and related animal and cell experiments.

In this study, our results showed an approximately independent positive correlation between FGF21 and age (*P* = 0.089), although this relationship had insufficient statistical significance which may due to the relatively small sample size. Previous study has indicated that serum FGF21 level was increased in population with obesity or diabetes [41]. However, FGF21 levels has no difference between HD patients with or without comorbidity of diabetes in our study. The discordance may be attributed to following factors: (1) Eun SH et al. found that serum FGF21 level was only increased to 1.5 times in patients with type 2 diabetes compared with that in healthy individuals [42]. While in our study, serum FGF21 level of HD patients was 3–5 times higher than that in control group and this huge increase may mask the impact of diabetes on FGF21; (2) the independent correlation between diabetes and FGF21 can also be diminished by the high incidence of secondary glucose and lipid metabolic disorders in HD patients.

Currently, DXA and CT are the main techniques to assess bone loss and diagnose osteoporosis in patients on HD in clinical practice, and they still have some disadvantages, such as exposure to radiation and high cost. Besides, the 2009 KDIGO Guideline recommended that BMD testing not be performed routinely in patients with CKD G3a to G5D [1]. In our study, we found that FGF21 had a great value in predicting osteoporosis with a relatively high sensitivity (80.2 %). Moreover, the sensitivity of predicting osteoporosis could rise to 91.4 % when FGF21 was combined with age. However, both of their specificities were below 50 %, which could be ascribed to the different residual renal function (RRF) in this research and complicated bone metabolism in CKD. Since serum FGF21 level is strongly dependent on RRF [20] that generally estimated by the 24-hour urine volume [43], we divided our osteoporosis HD patients into two subgroups based on the volume of 24-hour urine (24-hour urine volume **≤** 100 mL defined as no significant RRF [42]) and performed ROC analysis of FGF21 respectively to avoid relevant bias. As expected, the outstanding specificity (92.1 %) of FGF21 in HD patients with anuria proved our hypothesis. In contrast to FGF21, we observed that FGF23 had no value in predicting osteoporosis, although FGF23 had been proven to play a significant role in bone metabolism. The downside is that there was no difference in CT attenuation between the low and high level FGF21 subgroups in this study, this may due to the skewed distribution of FGF21 and it is difficult to find a suitable cut off value for the diagnosis of osteoporosis as a result. In general, either FGF21 alone or combined with age was an effective predictor of osteoporosis in patients on HD, especially those with less RRF. Our results can provide an easier way to identify patients with CKD with a high risk of osteoporosis and avoid extra radiation and cost. It may also provide a new therapeutic target for treating osteoporosis in patients on HD.

It must be admitted that our study also has some limitations: (1) the sample size in our study is relatively small, and more patients need to be enrolled to confirm the results; (2) other factors, such as 1,25-dihydroxyvitamin D and glucose, were not available in this study; (3) the association of FGF21 levels and DXA-based BMD levels were not explored; and (4) our results do not extend to other regions and ethnicities.

In conclusion, elevated FGF21 levels have a positive relationship with the incidence of osteoporosis in patients on HD. In addition, FGF21 alone or combined with age can be a predictive biomarker for osteoporosis in patients on HD, especially those with less RRF.

## Supplementary Information

**Additional file 1.**

## Data Availability

The datasets analysed during the current study are available from the corresponding author on reasonable request.
