# Supplementary material for: Fibroblast growth factor 21 (FGF21) is a sensitive marker of osteoporosis in haemodialysis patients: a cross-sectional observational study
Source: BMC Nephrol. 2021 May 19;22:183. doi: 10.1186/s12882-021-02393-z (PMC8135985; doi:10.1186/s12882-021-02393-z)
Supplement: Supplementary file 1 — Additional file 1. [file 12882_2021_2393_MOESM1_ESM.docx]

**Supplemental Table 1. Univariate logistic regression analysis for the association of FGF21 levels with CT attenuation values in patients on HD.**

| **Variables** | **Univariate analyze** | ***P* value** |
| --- | --- | --- |
|  | **Odds ration(95%CI)** |  |
| FGF21 tertile 1 | Ref | Ref |
| FGF21 tertile 2 | 0.750(0.454, 1.239) | 0.258 |
| FGF21 tertile 3 | 0.420(0.271, 0.651) | **<0.001**** |

**P*<0.05, ***P*<0.01.

FGF21, fibroblast growth factor 21.

**Supplemental Table 2.** **Multivariate logistic regression analysis for the association of age, dialysis vintage, DBP, FGF21, FGF23, major osteoporosis and hip fracture scores with CT attenuation values in patients on HD.**

| **Variables** | **Multivariate analyze** | ***P* value** |
| --- | --- | --- |
|  | **Odds ration(95%CI)** |  |
| Age(years) | 1.065(1.029, 1.103) | **<0.001**** |
| Dialysis vintage(years) | 1.020(0.962, 1.081) | 0.514 |
| DBP(mmHg) | 0.997(0.974, 1.019) | 0.762 |
| FGF21 (pg/ml) | 1.002(1.001, 1.003) | **<0.001**** |
| FGF23(pg/ml) | 1.000(1.000, 1.000) | 0.726 |
| MO scores | 0.842(0.628, 1.130) | 0.252 |
| HF scores | 1.298(0.847, 1.989) | 0.231 |

**P*<0.05, ***P*<0.01.

FGF21, fibroblast growth factor 21; FGF23, fibroblast growth factor 23; DBP, diastolic blood pressure; MO, major osteoporosis; HF, hip fracture.

**Supplemental Table 3.** **Multivariate linear regression analyses for the establishment of factors independently associated with FGF21.**

| **Variables** | **β（95% CI）** | ***P* value** |
| --- | --- | --- |
| Age | 0.092 | 0.089 |
| Dialysis duration | 0.065 | 0.240 |
| BMI | 0.013 | 0.799 |
| TG | -0.053 | 0.309 |
| TC | 0.055 | 0.296 |
| Ca | 0.796(0.111,1.482) | **0.023*** |
| Pi | 0.005 | 0.924 |
| iPTH | 0.000 | 0.994 |
| Alb | -0.062(-0.088,-0.035) | **0.000**** |
| Dicarbonate | -0.079(-0.121,-0.038) | **0.000**** |
| CVD | -0.048 | 0.362 |
| DM | -0.019 | 0.726 |
| HBP | -0.575(-1.020,-0.130) | **0.011*** |
| Ca usage | 0.039 | 0.460 |
| Vitamin D usage -0.005 | | 0.922 |

**P*<0.05, ***P*<0.01.

FGF21, fibroblast growth factor 21; BMI, body mass index; TC, total cholesterol; TG, triglyceride; Ca, calcium; Pi, phosphate; iPTH, intact parathyroid hormone; Alb, albumin; CVD, cardiocerebrovascular disease; DM, diabetes mellitus; HBP, high blood pressure.
